# Content Quality of Web-Based Short-Form Videos for Fire and Burn Prevention in China: Content Analysis

**DOI:** 10.2196/47343

**Published:** 2023-06-30

**Authors:** Lang Qin, Ming Zheng, David C Schwebel, Li Li, Peixia Cheng, Zhenzhen Rao, Ruisha Peng, Peishan Ning, Guoqing Hu

**Affiliations:** 1 Department of Epidemiology and Health Statistics, Hunan Provincial Key Laboratory of Clinical Epidemiology Xiangya School of Public Health Central South University Changsha China; 2 Nosocomial Infection Control Center Xiangya Hospital Central South University Changsha China; 3 Department of Psychology University of Alabama at Birmingham Birmingham, AL United States; 4 Department of Child, Adolescent and Women's Health School of Public Health Capital Medical University Beijing China; 5 National Clinical Research Center for Geriatric Disorders Xiangya Hospital Central South University Changsha China

**Keywords:** fire, burn, prevention, first aid, short video, content quality, public impact, China

## Abstract

**Background:**

Web-based short-form videos are increasingly popular for disseminating fire and burn prevention information, but their content quality is unknown.

**Objective:**

We aimed to systematically assess the characteristics, content quality, and public impact of web-based short-form videos offering primary and secondary (first aid) prevention recommendations for fires and burns in China between 2018 and 2021.

**Methods:**

We retrieved short-form videos offering both primary and secondary (first aid) information to prevent fire and burn injuries published on the 3 most popular web-based short-form video platforms in China: TikTok, Kwai, and Bilibili. To assess video content quality, we calculated the proportion of short-form videos that included information on each of the 15 recommendations for burn prevention education from the World Health Organization (WHO; *P*_1_) and that correctly disseminated each recommendation (*P*_2_). High *P*_1_ and *P*_2_ indicated better content quality. To assess their public impact, we calculated the median (IQR) of 3 indicators: the number of comments, likes, and saves as a favorite by viewers. Chi-square test, trend chi-square test, and Kruskal-Wallis *H* test examined differences in indicators across the 3 platforms, years, content, and time duration of videos and between videos disseminating correct versus incorrect information.

**Results:**

Overall, 1459 eligible short-form videos were included. The number of short-form videos increased by 16 times between 2018 and 2021. Of them, 93.97% (n=1371) were about secondary prevention (first aid) and 86.02% (n=1255) lasted <2 minutes. The proportion of short-form videos including each of the 15 WHO recommendations ranged from 0% to 77.86% (n=1136). Recommendations 8, 13, and 11 had the highest proportions (n=1136, 77.86%; n=827, 56.68%; and n=801, 54.9%, respectively), whereas recommendations 3 and 5 were never mentioned. Among the short-form videos that included the WHO recommendations, recommendations 1, 2, 4, 6, 9, and 12 were always disseminated correctly, but the other 9 recommendations were correctly disseminated in 59.11% (120/203) to 98.68% (1121/1136) of videos. The proportion of short-form videos including and correctly disseminating the WHO recommendations varied across platforms and years. The public impact of short videos varied greatly across videos, with a median (IQR) of 5 (0-34) comments, 62 (7-841) likes, and 4 (0-27) saves as a favorite. Short-form videos disseminating correct recommendations had larger public impact than those disseminating either partially correct or incorrect knowledge (median 5 vs 4 comments, 68 vs 51 likes, and 5 vs 3 saves as a favorite, respectively; all *P*<.05).

**Conclusions:**

Despite the rapid increase in the number of web-based short-form videos about fire and burn prevention available in China, their content quality and public impact were generally low. Systematic efforts are recommended to improve the content quality and public impact of short-form videos on injury prevention topics such as fire and burn prevention.

## Introduction

Fires and burns are the second-leading cause of injuries worldwide [1]. According to Global Burden of Disease estimates, fires and burns caused 111,292 deaths worldwide in 2019, resulting in the loss of 7,460,448 life-years [2]. In China, where this research was conducted, the number of deaths and disability-adjusted life years due to fires and burns were 11,095 persons and 687,955 years, respectively [2].

The implementation of high-quality safety education to the population is an important strategy to prevent fires and burns (primary prevention) and to reduce their adverse consequences following injury (secondary prevention) [3-5]. Despite public education efforts by multiple parties in China over many years [6], however, fewer than 40% of Chinese citizens report having basic knowledge of primary or secondary (first aid) prevention for fire and burn injuries (range of 6.3% to 39.2% across studies [7-9]). In 2021, the Pediatric Disaster Branch of the Chinese Pediatric Society of Chinese Medical Association published an expert consensus document that advocated the use of various channels to disseminate accurate injury prevention knowledge in an effective and engaging manner [10].

Web-based short-form videos offer one such strategy. They have emerged in recent years as a popular channel to deliver information publicly in China. According to a report from the China Internet Network Information Centre, the number of short-form video users jumped dramatically from 612 million in 2018 to 934 million in 2021, accounting for 90.5% of total internet users [11,12]. Accordingly, a substantial number of short-form videos on various topics, including fire and burn prevention, have been recently developed and disseminated on the internet.

One concern is that short-form videos about public health may disseminate inaccurate or misleading information. Unfortunately, relevant evidence concerning the content quality of short-form videos about fire and burn prevention in China is absent. To address this concern, this study was designed to systematically assess the characteristics, content quality, and public impact of web-based short-form videos about fire and burn prevention available in China. It extends the only previously published research on the topic, which considered English-language videos presented on YouTube and focused primarily on the physical properties of the videos (ie, the length of video, days included on the platform, and video producers) and physicians’ subjective evaluations of the video content [13,14]. Three key research questions were examined in this study: (1) How did the number of published short-form videos concerning fire and burn prevention change in China between 2018 and 2021? (2) Did the content of short-form videos for fire and burn prevention include and follow the World Health Organization (WHO) recommendations? and (3) Did incorrect content shown in short-form videos have an impact on the public?

## Methods

### Data Sources

We searched the 3 most popular web-based short-form video platforms in China—TikTok [15], Kwai [16], and Bilibili [17]—for eligible short-form videos. In 2020, the number of registered users on the 3 platforms were 447 million, 177 million, and 129 million, respectively, accounting for 45.2%, 17.9%, and 13% of all platform-registered users in China [18]. According to the platform statistics, TikTok had over 600 million daily active users on March 31, 2021 [19], and Kwai had over 308 million daily active users on December 31, 2021, on average [20]. Bilibili is a platform that publishes entertainment and educational videos [21] and had a comparatively low average number of daily active users—over 72.2 million on December 31, 2021 [22].

### Search Strategy

Following strategies used in previous research [23], we selected 7 search terms, including 2 core terms (fire and burn, namely “烧伤” and “烫伤” in Chinese) and 5 synonyms of those terms (“烧烫伤,” “受火焰烧灼,” “灼伤,” “烧残,” and “灼烧” in Chinese). Videos were eligible for inclusion if they met the following criteria: (1) disseminated advice for preventing fire and burn injuries, including both primary prevention (prevent injuries before they occur) and secondary prevention (reduce the impact of injuries after they occur); (2) focused on fire and burn injury prevention for humans rather than animals; and (3) had a total video length of <5 minutes. We limited our search to videos released between January 1, 2018, and December 31, 2021. In total, we searched 2498 videos concerning fire and burn prevention on TikTok, Kwai, and Bilibili. After removing 700 videos with invalid links and 339 duplicate videos, we finally included 1459 eligible short-form videos in this study.

### Evaluation of Content Quality and Public Impact of Short-Form Videos

We merged the WHO recommendations for fire and burn prevention from 2 professional reports [24,25]. After removing duplicate recommendations and those infeasible for the public to implement (eg, establishing smoke alarm laws and strictly enforcing them), we selected 15 items to evaluate in the videos: 6 focused on primary prevention (items 1-6) and 9 focused on secondary prevention or first aid (items 7-15; Table 1). All 15 items were scored in a binary fashion (yes or no) to evaluate whether the short-form video accurately disseminated the WHO-recommended prevention strategy with viewers.

We computed 2 proportions to assess the content quality of the short-form videos by measuring the inclusion and accuracy of disseminated content concerning each of the 15 WHO recommendations: (1) the proportion of short-form videos including the WHO recommendations (*P*_1_) and (2) the proportion of short-form videos correctly disseminating the content of the recommendations (*P*_2_). High *P*_1_ and *P*_2_ values indicated better content quality. These proportions were calculated as:




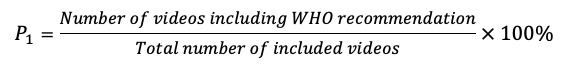







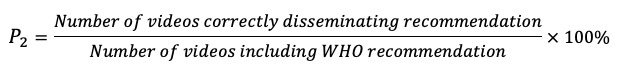




We assessed the public impact of the short-form videos by computing the median, IQR, and range of 3 indicators for each video: the number of comments, likes, and saves as a favorite by viewers. The distributions of all 3 indicators were positively skewed.

**Table 1:** The 15 recommendation items concerning fire and burn prevention.

Recommendation item	Description of item
1	Use of smoke detectors
2	Lowering the temperature of hot-water heaters
3	Installing sprinkler systems
4	Promoting the use of fire-retardant materials in the home for items such as mattresses, upholstered furniture, and pajamas
5	Promoting the use of flame-resistant sleepwear for children
6	Going to dedicated burn centers for burn treatment
7	Removing clothing to stop the burning process
8	Irrigating wounds with cold water for some time
9	Rolling on the ground, applying a blanket, or applying water or fire extinguishing liquids on active flame injuries
10	Removing or diluting the chemical agent by copiously irrigating wounds with water after chemical burns
11	Obtaining medical care
12	Do not commence first aid for victims before ensuring your own safety (eg, switch off electrical current and wear gloves for chemicals)
13	Avoiding the application of topical medication until the patient is placed under appropriate medical care
14	Do not apply ice to burn injuries
15	Do not open blisters with a needle or pin

### Data Collection

Using a web crawler algorithm developed on Python (version 3.0; Python Software Foundation), we automatically collected the full video content along with basic video descriptive information (title, published date, and URL) and public impact data (the number of comments, likes, and saves as a favorite by viewers). Duplicate and ineligible videos were removed by manually reviewing video titles, URLs, and full videos when needed.

All eligible videos were manually coded by trained researchers to determine if they included the 15 evaluation items. We randomly selected 10% of the included videos for double coding by 2 independent researchers. Consistency between the researchers ranged from 82.1% to 100% (overall 96.87%) across the 15 items.

### Statistical Analysis

Chi-square tests examined differences in the proportion of short-form videos that included and correctly disseminated each of the 15 WHO recommendations across the 3 platforms. Trend chi-square test examined changes in those proportions across years. Median, IQR, and range were used to describe the public impact indicators of the short-form videos. For each public impact indicator, Kruskal-Wallis *H* test compared differences across the platforms, years, content, and time duration of videos and between videos disseminating correct versus incorrect information about fire and burn prevention. All statistical analyses were performed using the SPSS software (version 25.0; IBM Corp). *P*<.05 in 2-tailed tests was considered statistically significant.

### Ethics Approval

This study was approved by the Ethics Committee of Xiangya School of Public Health (XYGW-2021-110). The study used open-access social media data and excluded all personal information and, therefore, was exempt from requiring informed consent.

## Results

### Characteristics of the Included Short-Form Videos

In total, 1459 eligible web-based short-form videos about fire and burn prevention were included. Of them, 409 (28.03%), 593 (40.65%), and 457 (31.32%) were from TikTok, Kwai, and Bilibili, respectively. The number of short-form videos increased by 16 times between 2018 and 2021, rising from 41 to 684 videos per year. Most videos presented secondary prevention strategies (n=1371, 93.97%) and had a time duration of <2 minutes (n=1255, 86.02%; Table 2).

**Table 2:** Characteristics of the 1459 included short-form videos about fire and burn prevention in China, 2018-2021.

Variable		Video (n=1459), n (%)
**Video platform**		
	TikTok	409 (28.03)
	Kwai	593 (40.65)
	Bilibili	457 (31.32)
**Year of video publication**		
	2018	41 (2.81)
	2019	164 (11.24)
	2020	578 (39.62)
	2021	676 (46.33)
**Content of video**		
	Primary prevention	74 (5.07)
	Secondary prevention (first aid)	1371 (93.97)
	Both primary and secondary prevention	14 (0.96)
**Time duration of video (minutes)**		
	<1	958 (65.66)
	1-1.9	297 (20.36)
	2-2.9	115 (7.88)
	3-3.9	61 (4.18)
	4-5	28 (1.92)

### Content Quality of Short-Form Videos

#### Proportion of Videos Including the WHO Recommendations

The proportion of the 1459 web-based short-form videos that included each of the 15 WHO recommendations for fire and burn injury prevention ranged from 0% to 77.86% (n=1136; Table 3). Of the 15 items, items 8, 13, and 11 were included in the highest proportions of videos, at 77.86% (n=1136), 56.68% (n=827), and 54.9% (n=801), respectively. Items 7, 15, and 14 were present in 36.33% (n=530), 16.93% (n=247), and 13.91% (n=203) of the videos, respectively, and the remaining 9 items appeared in less than 10% of the videos. Items 3 and 5 were not included in any of the videos at all.

The proportions of inclusion differed across the 3 short-form video platforms for items 6, 8, 9, 13, 14, and 15 (all *P*<.05). Three items showed change in inclusion over time: the proportion of videos including item 7 increased significantly from 2018 to 2021 (*P*=.004), and the proportions including items 8 and 13 decreased (*P*=.03 and *P*<.001, respectively; Table 3 and Table S1 in Multimedia Appendix 1).

**Table 3:** Proportion of short-form videos including the 15 fire and burn prevention recommendations in China, 2018-2021

Recommendation item	Video (n=1459), n (%)	Difference by platform			Change across years	
		*χ*^2^ (*df*)	*P* value	*χ*^2^_trend_ (*df*)		*P* value
1	1 (0.07)	1.98 (2)	.59	0.83 (3)		.36
2	1 (0.07)	1.98 (2)	.59	0.83 (3)		.36
3	0 (0)	—^a^	—^a^	—^a^		—^a^
4	2 (0.14)	1.90 (2)	.31	0.29 (3)		.59
5	0 (0)	—^a^	—^a^	—^a^		—^a^
6	8 (0.55)	6.31 (2)	.04	0.39 (3)		.54
7	530 (36.33)	4.31 (2)	.12	8.20 (3)		.004
8	1136 (77.86)	6.82 (2)	.03	4.75 (3)		.03
9	7 (0.48)	8.42 (2)	.005	0.90 (3)		.34
10	19 (1.3)	3.61 (2)	.17	1.15 (3)		.28
11	801 (54.9)	2.20 (2)	.33	0.91 (3)		.34
12	1 (0.07)	1.46 (2)	>.99	0.83 (3)		.36
13	827 (56.68)	13.84 (2)	.001	13.40 (3)		<.001
14	203 (13.91)	13.97 (2)	.001	0.10 (3)		.76
15	247 (16.93)	20.88 (2)	<.001	1.77 (3)		.18

^a^The chi-square test or trend chi-square test could not be performed because of the numerator had a value of zero.

#### Proportion of Videos Correctly Disseminating the WHO Recommendations

When present, items 1, 2, 4, 6, 9, and 12 were all disseminated correctly according to the WHO recommendations (Table 4). However, for items 13 and 14, the proportions correctly disseminating the WHO recommendations were just 62.64% (518/827) and 59.11% (120/203), respectively (Table 4).

The proportions of short-form videos correctly disseminating the WHO recommendations were statistically differently across the 3 web-based platforms for items 7, 8, 10, 11, and 13 (all *P*<.05). An analysis of change across years yielded 2 significant findings: the proportion of short-form videos correctly disseminating WHO recommendation item 11 decreased over time (*P*=.003), and the proportions correctly disseminating items 8 and 13 increased significantly across the study years (*P*=.04 and *P*<.001, respectively; Table 4 and Table S2 in Multimedia Appendix 1).

**Table 4:** Proportion of short-form videos correctly disseminating the 15 fire and burn prevention recommendations in China, 2018-2021.

Recommendation item	Video, n/N (%)	Difference by platform		Change across year	
		*χ*^2^ (*df*)	*P* value	*χ*^2^_trend_ (*df*)	*P* value
1	1/1 (100)	—^a^	—^a^	—^a^	—^a^
2	1/1 (100)	—^a^	—^a^	—^a^	—^a^
3	—^b^	—^a^	—^a^	—^a^	—^a^
4	2/2 (100)	—^a^	—^a^	—^a^	—^a^
5	—^b^	—^a^	—^a^	—^a^	—^a^
6	8/8 (100)	—^a^	—^a^	—^a^	—^a^
7	517/530 (97.55)	10.24 (2)	.006	0.11 (3)	.75
8	1121/1136 (98.68)	7.55 (2)	.02	4.11 (3)	.04
9	7/7 (100)	—^a^	—^a^	—^a^	—^a^
10	15/19 (78.95)	5.24 (2)	.04	0.13 (3)	.72
11	744/801 (92.88)	85.36 (2)	<.001	8.66 (3)	.003
12	1/1 (100)	—^a^	—^a^	—^a^	—^a^
13	518/827 (62.64)	27.99 (2)	<.001	22.26 (3)	<.001
14	120/203 (59.11)	4.01 (2)	.14	0.17 (3)	.68
15	221/247 (89.47)	3.96 (2)	.14	2.92 (3)	.09

^a^The chi-square test or trend chi-square test could not be performed because of the numerator had a value of zero.

^b^The proportion could not be calculated because of the denominator had a value of zero.

### Public Impact of Short-Form Videos

The median (IQR) of the 3 public impact indicators were 5 (0-34) comments, 62 (7-841) likes, and 4 (0-27) saves as a favorite by viewers. The 3 public impact indicators all differed significantly across platforms and across years (all *P*<.05). Videos about both primary and secondary prevention had a greater number of likes and saves as a favorite than those focusing on only primary or only secondary (first aid) prevention. Videos with a time duration of <2 minutes had more comments and likes (Table 5 and Table S3 in Multimedia Appendix 1).

**Table 5:** Public impact of short-form videos for fire and burn prevention in China, 2018-2021.

Public impact indicator	Value, median (IQR; range)	Difference by platform			Difference across years			Difference by video content			Difference by time duration	
		*H*	*P* value	*H*		*P* value	*H*		*P* value	*H*		*P* value
Number of comments per video	5 (0-34; 0-62,000)	462.62	<.001	23.72		<.001	1.25		.54	31.69		<.001
Number of likes per video	62 (7-841; 0-18,390,000)	617.06	<.001	38.64		<.001	14.56		<.001	35.88		<.001
Number of saves as a favorite per video	4 (0-27; 0-34,000)	323.86	<.001	24.84		<.001	7.73		.02	2.03		.85

### Association of Content Quality of Short-Form Videos With Their Public Impact

Compared to short-form videos that incorrectly disseminated the WHO recommendations, videos that correctly disseminating the WHO recommendations had significantly higher public impact: median 5 versus 4 comments (*P*=.003), 68 versus 51 likes (*P*=.001), and 5 versus 3 saves as a favorite (*P*<.001), respectively (Table 6).

**Table 6:** Public impact differences between videos correctly disseminating the World Health Organization recommendations and those that do not in China, 2018-2021.

Public impact indicator and type of video		Value, median (IQR; range)	*H*		*P* value	
**Number of comments per video**				8.72		.003
	Correct dissemination	5 (0-47; 0-62,000)				
	Incorrect dissemination	4 (1-18; 0-13,000)				
**Number of likes per video**				11.21		.001
	Correct dissemination	68 (7-1345; 0-1,861,000)				
	Incorrect dissemination	51 (7-270; 0-18,390,000)				
**Number of saves as a favorite per video**				15.15		<.001
	Correct dissemination	5 (1-40; 0-34,000)				
	Incorrect dissemination	3 (0-15; 0-7006)				

## Discussion

### Principal Findings

This study systematically assessed the characteristics (video platform, year of publication, content, and time duration), content quality, and public impact of 1459 web-based short-form videos about fire and burn prevention in China. Three key findings emerged. First, the number of short-form videos concerning fire and burn prevention increased by 16 times between 2018 to 2021 in China. Second, the 15 WHO recommendations concerning fire and burn prevention were seldom included in published Chinese web-based short-form videos, and 9 of the 15 recommendations were not correctly disseminated by most or all of the 1459 short-form videos. Third, the short-form videos disseminating accurate fire and burn prevention recommendations had larger public impact than those disseminating partially or fully incorrect information.

### Interpretation

#### Characteristics of Short-Form Videos

The striking increase in the number of short-form videos appearing on the web about fire and burn prevention between 2018 and 2021 is likely associated with the increase in short-form video viewers in China over that time period (from 612 million to 926 million) and the average daily time viewing short-form videos among those users (from 88 minutes to 125 minutes) [12,26]. Increased viewership is explained by the multiple advantages of short-form videos as a tool to share information, including their easy-to-understand, plentiful content and their free and easy web-based access at any time and place [26,27].

#### Content Quality of Short-Form Videos

Building from previous studies that relied on reviewers’ judgment to assess the content quality of short English-language videos on YouTube [13,14], this study systematically evaluated the content quality of web-based short-form videos about fire and burn prevention in China. Our results discovered that 11 of the 15 WHO recommendations were excluded by over 80% of short-form videos, and 2 items (item 3 “installing sprinkler systems” and item 5 “promoting use of children’s flame-resistant sleepwear”) were omitted completely by all included videos. The proportions of short-form videos correctly disseminating information concerning item 13 (“avoiding application of topical medication prior to medical care”) and item 14 (“not applying ice to burn injuries”) were also low.

We conclude that the content quality of the web-based videos was generally low. Incorrect and missing content may be explained because short-form video developers, web-based platform supervisors, and relevant government officials are unaware of the recommended injury prevention strategies [27] and lack oversight to ensure the content of videos are delivered with accurate and thorough information [13,19,28,29]. Significant differences in the proportion of included and correct information delivered across platforms likely reflects a mix of inconsistent proficiency in platform operation and supervisor, differences in the type of video developer who typically posts videos to specific platforms, and differences in the typical audiences of the platforms [30]. For example, the Bilibili video platform includes a specific section for educational entertainment and therefore attracts professional video developers who have a stronger medical background. Bilibili also implements strict regulations and restrictions for videos published on their platform, adhering to national regulations on the management of network information. In contrast, TikTok and Kwai do not adhere as closely to national regulations [21,31].

Extending previous research that focused on the quality of short-form videos released in a single year [19,32,33], we examined change over time and found that the proportions of short-form videos including 12 of the 15 WHO recommendations did not significantly change between 2018 and 2021. The low and unchanging proportions of short-form videos including most of the 15 WHO recommendations between 2018 and 2021 suggest that video developers, platform managers, and governmental agency supervisors did not work to improve the delivery of fire and burn injury prevention videos over recent years in China.

#### Public Impact of Short-Form Videos

Compared to short-form videos targeting other health topics [28,34,35], the web-based short-form videos about fire and burn prevention that we examined were generally less frequently commented on, given likes, or saved as favorites. The low public impact may be related to the attractiveness of content. It also may be related to the low quality of published videos, including the accuracy of content and the artful production of the videos [34,36,37].

The differences in public impact across platforms and years likely reflect the number of users registered across the 3 platforms and over time [11,12], as well as increasing public awareness for and interest in fire and burn prevention aroused by highly publicized fire and burn injury events in China recently [38].

### Implications

To effectively prevent fire and burn injuries and mitigate the severity of injuries after they occur, thorough and accurate primary and secondary prevention knowledge should be disseminated to the public in an effective and engaging manner. Web-based short-form videos offer an excellent strategy to deliver that information. We recommend 3 strategies to improve the content quality and public impact of web-based short-form videos about fire and burn prevention in China. First, the Chinese government should release regulations requiring both web-based video platforms and video developers to adhere to the WHO recommendations when designing and posting short-form videos on the prevention of fire and burn injuries, as well as videos on the prevention of other types of injuries and diseases.

Second, routine monitoring and supervision should be strictly implemented by the government to detect videos that disseminate incorrect information. The 15 recommendations that we considered in this study could be used to assess the content quality of a massive number of short-form videos concerning fire and burn prevention rapidly and quantitatively. Videos flagged for disseminating falsehoods should be removed from the platform immediately upon detection. Platforms that are found to frequently disseminate inaccurate prevention videos should be required to improve their supervision processes or be closed if they fail to make change.

Finally, multidisciplinary teams should be organized to produce high-quality short-form videos about fire and burn prevention, as well as the prevention and control of other common injuries and diseases. These videos should be produced in an engaging and educational manner that appeals to the target audiences and disseminates thorough and accurate knowledge.

### Limitations

This study has several limitations. First, we only retrieved short-form videos published on the 3 most popular web-based video platforms in China, and therefore, we likely omitted eligible videos posted on other platforms. We deemed it unlikely that the inclusion of videos from other platforms would significantly affect our results since (1) other short-form video platforms have small numbers of registered users and rarely develop and publish original videos, instead reposting videos from the 3 platforms we included in this study [39,40], and (2) nearly all video developers, platform managers, and governmental department video supervisory officials are uneducated in the WHO injury prevention strategies, and thus, all videos posted on any web-based platform in China are likely to show similar patterns [27,41].

A second study limitation is that our findings may be affected somewhat by subjective perceptions of the researchers while coding the 15 WHO recommendations. However, all raters received standardized training and they demonstrated excellent consistency coefficients when coding a randomly selected 10% sample of the 1459 videos. Last, this study did not evaluate other attributes of the short-form videos’ quality, including more subjective assessment of the quality of the fire and burn prevention advice provided or assessment from the perspectives of communication theory or artistic presentation.

### Conclusions

The number of short-form videos about fire and burn prevention posted on web-based Chinese platforms increased rapidly over the past few years, but many videos did not include correct information recommended by the WHO concerning basic primary or secondary prevention knowledge. Many videos had low public impact. The dissemination of accurate prevention information was associated with comparatively larger public impact.

Based on these findings, we recommend systematic efforts to improve the content quality and public impact of short-form videos about fire and burn prevention in China, thus yielding substantial public health benefits by improving basic knowledge of fire and burn prevention among citizens. These efforts should include obligatory professional training for video developers, platform managers, and governmental departmental supervisors; strict and careful review of all videos posted to web-based platforms; and strict governmental review and supervision of the content of short-form videos published on Chinese internet platforms.

## Appendix

Multimedia Appendix 1 Proportion of short-form videos including recommendations by platform and year; proportion of short-form videos correctly disseminating recommendations by platform and year; and public impact of short-form videos by platform, year, content, and time duration.

## Abbreviations

WHO: World Health Organization

## Data Availability

The data sets generated and analyzed during this study are available from the corresponding author on reasonable request. Interested parties will be required to complete an institutional Data Use Agreement, and data will be made available via Secure Data Transfer.
