# Supplementary material for: Content Quality of Web-Based Short-Form Videos for Fire and Burn Prevention in China: Content Analysis
Source: J Med Internet Res. 2023 Jun 30;25:e47343. doi: 10.2196/47343 (PMC10365633; doi:10.2196/47343)
Supplement: Multimedia Appendix 1 [file jmir_v25i1e47343_app1.docx]

**Appendix tables**

1. Appendix Table S1. Proportion of short videos including 15 fire/burn prevention recommendations by platform and across years in China, 2018-2021

2. Appendix Table S2. Proportion of short videos correctly disseminating 15 fire/burn prevention recommendations by platform and across years in China, 2018-2021

3. Appendix Table S3. Public impact of fire/burn prevention short videos by platform, years, content, and time duration in China, 2018-2021

**Appendix Table S1.** Proportion of short videos including 15 fire/burn prevention recommendations by platform and across years in China, 2018-2021

| **Recommendation** | **Proportion by platform** | | | | |  | **Proportion across years** | | | | | |
| --- | --- | --- | --- | --- | --- | --- | --- | --- | --- | --- | --- | --- |
|  | **TikTok** | **Kwai** | **Bilibili** | ***ꭓ*^2^** | ***P*-value** |  | **2018** | **2019** | **2020** | **2021** | ***ꭓ*^2^_trend_** | ***P*-value** |
| Item 1 | 0.00 | 0.00 | 0.22 | 1.98 | .60 |  | 0.00 | 0.00 | 0.00 | 0.15 | 0.83 | .36 |
| Item 2 | 0.00 | 0.00 | 0.22 | 1.98 | .60 |  | 0.00 | 0.00 | 0.00 | 0.15 | 0.83 | .36 |
| Item 3 | 0.00 | 0.00 | 0.00 | —^a^ | —^a^ |  | 0.00 | 0.00 | 0.00 | 0.00 | —^a^ | —^a^ |
| Item 4 | 0.00 | 0.34 | 0.00 | 1.88 | .31 |  | 0.00 | 0.00 | 0.35 | 0.00 | 0.29 | .59 |
| Item 5 | 0.00 | 0.00 | 0.00 | —^a^ | —^a^ |  | 0.00 | 0.00 | 0.00 | 0.00 | —^a^ | —^a^ |
| Item 6 | 0.00 | 1.18 | 0.22 | 6.31 | .04 |  | 0.00 | 0.61 | 0.87 | 0.30 | 0.39 | .54 |
| Item 7 | 39.12 | 33.22 | 37.86 | 4.31 | .12 |  | 29.27 | 23.78 | 37.89 | 38.46 | 8.20 | .004 |
| Item 8 | 78.00 | 74.87 | 81.62 | 6.82 | .03 |  | 82.93 | 78.66 | 80.97 | 74.70 | 4.75 | .03 |
| Item 9 | 0.00 | 1.18 | 0.00 | 8.42 | .005 |  | 0.00 | 0.00 | 0.52 | 0.59 | 0.90 | .34 |
| Item 10 | 2.20 | 1.01 | 0.88 | 3.61 | .17 |  | 0.00 | 1.83 | 1.90 | 0.74 | 1.15 | .28 |
| Item 11 | 55.75 | 56.49 | 52.08 | 2.20 | .33 |  | 60.98 | 55.49 | 55.71 | 53.70 | 0.91 | .34 |
| Item 12 | 0.00 | 0.17 | 0.00 | 1.46 | >.99 |  | 0.00 | 0.00 | 0.00 | 0.15 | 0.83 | .36 |
| Item 13 | 50.12 | 61.89 | 57.33 | 13.84 | .001 |  | 63.41 | 65.85 | 59.34 | 51.78 | 13.40 | <.001 |
| Item 14 | 10.02 | 12.98 | 18.60 | 13.970 | .001 |  | 19.51 | 8.54 | 15.22 | 13.76 | 0.10 | .76 |
| Item 15 | 16.87 | 12.31 | 22.98 | 20.878 | <.001 |  | 31.71 | 15.24 | 17.30 | 16.12 | 1.77 | .18 |

^a^: The chi-square test or trend chi-square test could not be performed because of a zero value of numerator.

**Appendix Table S2.** Proportion of short videos correctly disseminating 15 fire/burn prevention recommendations by platform and across years in China, 2018-2021

| **Recommendation** | **Proportion by platform** | | | | |  | **Proportion across years** | | | | | |
| --- | --- | --- | --- | --- | --- | --- | --- | --- | --- | --- | --- | --- |
|  | **TikTok** | **Kwai** | **Bilibili** | ***ꭓ*^2^** | ***P*-value** |  | **2018** | **2019** | **2020** | **2021** | ***ꭓ*^2^_trend_** | ***P*-value** |
| Item 1 | —^a^ | —^a^ | 100.00 | —^b^ | —^b^ |  | —^a^ | —^a^ | —^a^ | 100.00 | —^b^ | —^b^ |
| Item 2 | —^a^ | —^a^ | 100.00 | —^b^ | —^a^ |  | —^a^ | —^a^ | —^a^ | 100.00 | —^b^ | —^b^ |
| Item 3 | —^a^ | —^a^ | —^a^ | —^b^ | —^b^ |  | —^a^ | —^a^ | —^a^ | —^a^ | —^b^ | —^b^ |
| Item 4 | —^a^ | 100.00 | —^a^ | —^b^ | —^b^ |  | —^a^ | —^a^ | 100.00 | —^a^ | —^b^ | —^b^ |
| Item 5 | —^a^ | —^a^ | —^a^ | —^b^ | —^b^ |  | —^a^ | —^a^ | —^a^ | —^a^ | —^b^ | —^b^ |
| Item 6 | —^a^ | 100.00 | 100.00 | —^b^ | —^b^ |  | —^a^ | 100.00 | 100.00 | 100.00 | —^b^ | —^b^ |
| Item 7 | 98.13 | 94.92 | 100.00 | 10.24 | .006 |  | 100.00 | 97.44 | 96.80 | 98.08 | .11 | .75 |
| Item 8 | 100.00 | 97.97 | 98.39 | 7.55 | .02 |  | 94.12 | 97.67 | 98.93 | 99.01 | 4.11 | .04 |
| Item 9 | —^a^ | 100.00 | —^a^ | —^b^ | —^b^ |  | —^a^ | —^a^ | 100.00 | 100.00 | —^b^ | —^b^ |
| Item 10 | 100.00 | 50.00 | 75.00 | 5.24 | .04 |  | —^a^ | 66.67 | 81.82 | 80.00 | 0.13 | .72 |
| Item 11 | 100.00 | 82.99 | 100.00 | 85.36 | <.001 |  | 100.00 | 72.53 | 97.52 | 93.39 | 8.66 | .003 |
| Item 12 | —^a^ | 100.00 | —^a^ | —^b^ | —^b^ |  | —^a^ | —^a^ | —^a^ | 100.00 | —^b^ | —^b^ |
| Item 13 | 75.61 | 53.68 | 65.10 | 27.99 | <.001 |  | 65.38 | 34.26 | 64.72 | 69.14 | 22.26 | <.001 |
| Item 14 | 56.10 | 51.95 | 67.06 | 4.01 | .14 |  | 75.00 | 57.14 | 57.95 | 59.14 | 0.17 | .68 |
| Item 15 | 95.65 | 86.30 | 87.62 | 3.96 | .14 |  | 100.00 | 88.00 | 93.00 | 85.32 | 2.92 | .09 |

^a^: The proportion could not be calculated because of a zero value of denominator.

^b^: The chi-square test or trend chi-square test could not be performed because of a zero value of numerator.

**Appendix Table S3.** Public impact of fire/burn prevention short videos by platform, years, content, and time duration in China, 2018-2021

| **Variable** | **Number of comments per video** | | | | |  | **Number of likes per video** | | | | |  | **Number saved as a favorite per video** | | | | |
| --- | --- | --- | --- | --- | --- | --- | --- | --- | --- | --- | --- | --- | --- | --- | --- | --- | --- |
|  | ***M*** | ***IQR* (*P*_25_, *P*_75_)** | ***Range*** | ***H*** | ***P*** |  | ***M*** | ***IQR* (*P*_25_, *P*_75_)** | ***Range*** | ***H*** | ***P*** |  | ***M*** | ***IQR* (*P*_25_, *P*_75_)** | ***Range*** | ***H*** | ***P*** |
| **Video platform** | | |  |  |  |  |  |  |  |  |  |  |  |  |  |  |  |
| TikTok | 53 | 195 (15, 210) | 62000 | 462.62 | <.001 |  | 1401 | 8323 (411, 8734) | 1593999 | 617.06 | <0.01 |  | 47 | 214 (10, 224) | 34000 | 323.86 | <.001 |
| Kwai | 5 | 19 (1, 20) | 51000 |  |  |  | 68 | 290 (24, 314) | 18390000 |  |  |  | 3 | 13 (0, 13) | 26000 |  |  |
| Bilibili | 0 | 2 (0, 2) | 845 |  |  |  | 3 | 8 (1, 9) | 327000 |  |  |  | 1 | 5 (0, 5) | 2101 |  |  |
| **Year of video publication** | | |  |  |  |  |  |  |  |  |  |  |  |  |  |  |  |
| 2018 | 2 | 8 (1, 9) | 2498 | 23.72 | <.001 |  | 6 | 118 (4, 122) | 205000 | 38.64 | <0.01 |  | 4 | 22 (1, 23) | 15000 | 24.84 | <.001 |
| 2019 | 6 | 26 (1, 27) | 16000 |  |  |  | 142 | 600 (19, 619) | 634000 |  |  |  | 7 | 30 (1, 31) | 25000 |  |  |
| 2020 | 7 | 64 (1, 65) | 51000 |  |  |  | 150 | 1422 (7, 1429) | 1861000 |  |  |  | 7 | 42 (1, 43) | 26000 |  |  |
| 2021 | 3 | 22 (0, 22) | 62000 |  |  |  | 38 | 383 (5, 388) | 18390000 |  |  |  | 2 | 17 (0, 17) | 34000 |  |  |
| **Content of video** |  |  |  |  |  |  |  |  |  |  |  |  |  |  |  |  |  |
| Primary prevention | 8 | 6 (1, 8) | 918 | 1.25 | .54 |  | 41 | 226 (14, 240) |  | 14.56 | <.001 |  | 2 | 7 (0, 7) | 2 | 7.73 | .02 |
| Secondary prevention (first-aid) | 4 | 35 (0, 35) | 62000 |  |  |  | 57 | 836 (6, 842) |  |  |  |  | 4 | 27 (0, 27) | 4 |  |  |
| Both primary and secondary prevention | 8 | 96 (2, 98) | 690 |  |  |  | 477 | 2439 (144,2583) |  |  |  |  | 2 | 54 (1, 55) | 2 |  |  |
| **Time duration of video (minutes)** | |  |  |  |  |  |  |  |  |  |  |  |  |  |  |  |  |
| <1 | 6 | 37 (1, 38) | 62000 | 31.69 | <.001 |  | 96 | 874 (14, 888) | 18390000 | 35.88 | <.001 |  | 4 | 28 (0, 28) | 25000 | 2.03 | .845 |
| 1~ | 3 | 37 (0, 37) | 51000 |  |  |  | 48 | 911 (4, 915) | 1861000 |  |  |  | 4 | 29 (1, 30) | 26000 |  |  |
| 2~ | 2 | 24 (0, 24) | 31000 |  |  |  | 12 | 661 (3, 664) | 759000 |  |  |  | 6 | 23 (1, 24) | 18000 |  |  |
| 3~ | 1 | 15 (0, 15) | 2855 |  |  |  | 21 | 196 (4, 200) | 33000 |  |  |  | 4 | 26 (1, 27) | 922 |  |  |
| 4~5 | 1 | 13 (0, 13) | 3991 |  |  |  | 4 | 60 (1, 61) | 228000 |  |  |  | 4 | 13 (0, 13) | 34000 |  |  |
